# Joint Microbiota Activity and Dietary Assessment through Urinary Biomarkers by LC-MS/MS

**DOI:** 10.3390/nu15081894

**Published:** 2023-04-14

**Authors:** Victoria Ramos-Garcia, Isabel Ten-Doménech, Alba Moreno-Giménez, Laura Campos-Berga, Anna Parra-Llorca, María Gormaz, Máximo Vento, Melina Karipidou, Dimitrios Poulimeneas, Eirini Mamalaki, Eirini Bathrellou, Julia Kuligowski

**Affiliations:** 1 Neonatal Research Unit, Health Research Institute Hospital La Fe, Avda Fernando Abril Martorell 106, 46026 Valencia, Spain; victoria_ramos@iislafe.es (V.R.-G.); isabel_ten@iislafe.es (I.T.-D.); albamorenoo@hotmail.com (A.M.-G.); lauracamposberga@gmail.com (L.C.-B.); annaparrallorca@gmail.com (A.P.-L.); maximo.vento@uv.es (M.V.); 2 Division of Neonatology, University & Polytechnic Hospital La Fe, Avda Fernando Abril Martorell 106, 46026 Valencia, Spain; gormaz_mar@gva.es; 3 Department of Nutrition and Dietetics, Harokopio University of Athens, El. Venizelou 70, 17676 Kallithea, Greece; mkarip@hua.gr (M.K.); dpoul@hua.gr (D.P.); eir.mamalaki@gmail.com (E.M.); ebathrellou@hua.gr (E.B.)

**Keywords:** nutrition biomarkers, microbiota biomarkers, food-intake, dietary assessment, lactating mothers, urine

## Abstract

Accurate dietary assessment in nutritional research is a huge challenge, but essential. Due to the subjective nature of self-reporting methods, the development of analytical methods for food intake and microbiota biomarkers determination is needed. This work presents an ultra-high performance liquid chromatography coupled to tandem mass spectrometry (UHPLC-MS/MS) method for the quantification and semi quantification of 20 and 201 food intake biomarkers (BFIs), respectively, as well as 7 microbiota biomarkers applied to 208 urine samples from lactating mothers (M) (*N* = 59). Dietary intake was assessed through a 24 h dietary recall (R24h). BFI analysis identified three distinct clusters among samples: samples from clusters 1 and 3 presented higher concentrations of most biomarkers than those from cluster 2, with dairy products and milk biomarkers being more concentrated in cluster 1, and seeds, garlic and onion in cluster 3. Significant correlations were observed between three BFIs (fruits, meat, and fish) and R24h data (r > 0.2, *p*-values < 0.01, Spearman correlation). Microbiota activity biomarkers were simultaneously evaluated and the subgroup patterns detected were compared to clusters from dietary assessment. These results evidence the feasibility, usefulness, and complementary nature of the determination of BFIs, R24h, and microbiota activity biomarkers in observational nutrition cohort studies.

## 1. Introduction

The measurement of diet exposure is crucial for determining associations between food intake and health status. Additionally, optimizing nutritional advice to specific population groups (e.g., chronic diseases patients, lactating mothers, etc.) has recently become a major challenge [1]. The incidence of preterm deliveries (<37 weeks of gestation) and the survival rate of preterm infants have been steadily increasing over recent decades [2] and early infant nutrition is key for improving clinical outcomes. As human milk (HM) is recommended as the gold standard for infant nutrition [3] and the impact of maternal nutrition on the composition of HM has been evidenced [4,5,6], it is important to develop tools that can help to evaluate maternal dietary patterns with the aim of providing dietary advice that could enhance preterm infants’ growth rates.

Dietary intake data is most commonly collected using tools based on self-reporting, such as food frequency questionnaires (FFQ) for the assessment of regular consumption and food diaries or 24 h recalls (R24h) for the assessment of short-term consumption. This food intake data is then translated into quantitative information regarding specific nutrients or food groups using food composition databases. However, such methods are prone to errors due to their subjective nature [7]. In particular, foods perceived as being unhealthy (e.g., processed foods and high fats) are typically under-reported [8], while foods perceived as being healthy (e.g., fruits and vegetables) are often over-reported. Therefore, the generation of robust data on regular dietary intake is essential to improve the accuracy of dietary assessment. The measurement of metabolomic food intake biomarkers (BFIs) in biological samples [9,10] has emerged in recent years as a method of supporting a more accurate assessment of nutritional intake [11,12]. Metabolomic fingerprinting has opened new opportunities for the discovery of specific BFIs in body fluids [13]. For example, flavonoids, methyl histidine, isoflavones, trimethylamine N-oxide (TMAO), and arylglycines have been described recently as biomarkers of fruits and vegetables, meat, seeds, fish, and dairy products, respectively [10,14].

After ingestion, these compounds are metabolized by phase I and II enzymes, yielding glucuronidated, sulfated, and methylated metabolites [11]. Although recent studies illustrated that an individual’s dietary habits can predict the levels of specific metabolites present in plasma [15] and research has identified hundreds of mediation linkages that provide insight into diet—microbiome interactions [16], urine is the most practical and feasible biofluid used for BFI identification since many metabolic byproducts are excreted through it [17]. Compared to the collection of other biofluids, such as blood and plasma, the collection of urine is easier, cheaper, allows quicker collection of large volumes, and is less burdensome and invasive for the participants [18].

Due to the different nature of BFIs, high sensitivity and wide coverage analytical methods are needed to assess the metabolic fingerprint of food intake. Liquid (LC) or gas (GC) chromatography coupled with mass spectrometry (MS) and H^1^ nuclear magnetic resonance (NMR) spectroscopy are the most commonly used techniques for the analysis of BFIs [19] and targeted assays that enable the quantification of metabolites are the preferred strategy [20]. The vast majority of previously published studies about BFI fingerprinting required complex and time-consuming sample preparation, such as enzymatic hydrolysis [21,22] or solid phase extraction [23,24], resulting in incomplete hydrolysis and low recoveries, respectively. Moreover, the lack of commercial standards of metabolic byproducts entangle the targeted approach [25] and therefore an attractive strategy to overcome this drawback could be the semi-quantification of these urinary metabolic byproducts.

In addition, diet is one of the key factors involved in shaping the gut microbiota, affecting the microbial diversity, as well as the abundance of specific microbes [26,27,28]. Metabolic end products of dietary micro and macronutrients, such as bile acids (e.g., cholic and lithocholic acids), short chain fatty acids (e.g., acetic and butyric acid), indole and polyphenyl derivatives (e.g., 3-indolepropionic acid (3-IPA) and hippuric acid), and phenolic acids (e.g., gallic acid and ferulic acid) are used to assess the activity of gut microbiota [29,30,31,32,33]. A joint assessment of food intake and microbiota activity biomarkers would be of interest.

The aim of this work was to develop an ultra-high performance LC coupled with the tandem MS (UHPLC-MS/MS) method for the quantification and semi-quantification of 20 and 201 BFIs, respectively, of different food groups (e.g., fruits and vegetables, meat, etc.) and the simultaneous determination of 7 microbiota biomarkers. The applicability of the method was evaluated by the analysis of 208 urine samples from lactating mothers (*N* = 59). The performance of the determined BFIs as a complementary tool to self-reported R24h dietary assessment was evaluated. Furthermore, nutrition patterns were compared to microbiota activity biomarker diversity clusters.

## 2. Materials and Methods

### 2.1. Study Design, Population, and Sample Collection

Samples were collected under the framework of the NUTRISHIELD project (https://nutrishield-project.eu/ (accessed on 13 April 2023)), in a prospective, observational, cohort study (NCT05646940) including infant–mother dyads and performed at the Division of Neonatology of the University and Polytechnic Hospital La Fe (Valencia, Spain). Urine samples from lactating mothers were collected at six time points, covering the period from birth to six months of age. R24h were recorded at all timepoints except at birth, while FFQs were recorded at month one. During data analysis, urine samples and R24h from each time point were treated individually, as maternal food intake varied between time points. Demographic and clinical characteristics of participants are shown in Table 1. The mothers’ first morning urine was collected in sterile polypropylene PP containers. In total, 208 urine samples from 59 lactating mothers were collected. For additional information on the NUTRISHIELD study, the reader is referred to Ramos-Garcia et al. [34].

The study was approved by the Ethics Committee for Biomedical Research of the Health Research Institute La Fe, University and Polytechnic Hospital La Fe (Valencia, Spain) with registry #2019-289-1 and all methods were performed in accordance with the relevant guidelines and regulations. Written informed consents were obtained from lactating mothers prior to sample collection and analysis of clinical and demographic information.

### 2.2. Dietary Assessment

Regarding the dietary assessment methods, a R24h and a validated FFQ [35] were performed. For the R24h collection, trained researchers asked for all foods and beverages participants consumed the previous day, using the multiple-pass method [36]. Recall data were analyzed in terms of nutrients using the dietary analysis software Nutritionist Pro™ (2007, Axxya Systems, Redmond, WA, USA; https://nutritionistpro.com/). Additionally, dietary intake was grouped into food groups, namely fruits, vegetables, meat, fish, egg, bread/starch, seeds, milk, dairy products, fat, other carbohydrates, and soft drinks.

Regarding the FFQ, it comprises 142 questions on the consumption of foods that are commonly eaten by the Spanish population throughout the year, including dairy products, cereals, fruits, vegetables, meat, fish, legumes, added fats, alcoholic beverages, stimulants, and sweets. Using a 9-grade scale (“never or less than 1 time/month”, “1–3 times/month”, “1 time/week”, “3–4 times/week”, “5–6 times/week”, “1 time/day”, “2–3 times/day”, “4–5 times/day”, “≥6 times/day”) participants were required to indicate the absolute frequency of consuming a certain amount of food, expressed in g, milliliters or in other common measures, such as slice, tablespoon or cup, depending on the food. The previous month was set as the timeframe.

Based on the FFQ-responses, adherence to the Mediterranean diet was evaluated by using the MedDietScore, a composite score calculated for each participant [37]. For food groups presumed to be part of the Mediterranean pattern (i.e., those with a recommended intake of 4 servings per week or more, such as non-refined cereals, fruits, vegetables, legumes, olive oil, fish, and potatoes), higher scores are assigned when the consumption is according to the rationale of the Mediterranean pattern, while lower scores are assigned when participants report no, rare, or moderate consumption. For the consumption of foods presumed to be eaten less frequently within the Mediterranean diet (i.e., consumption of meat and meat products, poultry, and full fat dairy products), scores are assigned on a reverse scale. As the sample of the study is lactating mothers, the original score was modified by removing the component regarding alcohol consumption. Thus, the range of this modified MedDietScore is between 0 and 50, with higher values of the score indicating greater adherence to the Mediterranean diet.

### 2.3. Standards and Reagents

HPLC grade acetonitrile (ACN) (≥99.9%), ammonium formate (≥99.0%), formic acid (≥95%), phenylpropionylglycine (PPG) (≥99%), 3-indolepropionic acid (3-IPA) (≥99%), L-kynurenine (≥98%), 3-indoleacetic acid (3-IAA) (≥98%), hippuric acid (≥98%), ferulic acid sulphate (≥99%), proline betaine (≥99%), hesperetin (≥95%), phloretin (≥99%), quercetin (≥95%), kaempferol (≥97%), O-desmethylangolensin (O-DMA) (≥97%), daidzein (≥98%), equol (≥99%), glycitein (≥97%), genistein (≥98%), trimethylamine N-oxide (TMAO) (≥95%), isovalerylglycine (≥97%), isobutyrylglycine (≥95%), galactitol (≥99%), gallic acid (≥98%), and the “Amino Acid Standards, physiological” solution containing the amino acids L-tyrosine, 1-methylhistidine, 3-methylhistidine, anserine, citrulline, and taurine, as well as the isotopically labelled internal standards (IS) caffeine-D_9_ (≥99%), phenylalanine-D_5_ (≥99%), and taxifolin (≥95%) were purchased from Sigma-Aldrich Química SL (Madrid, Spain). Betaine-D_11_ (≥99%) was purchased from Cambridge Isotope Laboratories, Inc. (Tewksbury, MA, USA). Standard solutions were prepared in ultrapure water (Q-POD^®^ system, Merck KGaA, Darmstadt, Germany).

### 2.4. UHPLC-MS/MS Determination of Nutrition and Microbiota Biomarkers

The UHPLC-MS/MS method for the quantification of 20 nutrition biomarkers (i.e., proline betaine, hesperetin, phloretin, quercetin, kaempferol, O-DMA, daidzein, equol, glycitein, genistein, 1-methylhistidine, 3-methylhistidine, anserine, TMAO, isovalerylglycine, isobutyrylglycine, galactitol, gallic acid, citrulline, and taurine) and 7 microbiota biomarkers (i.e., PPG, 3-IPA, L-kynurenine, 3-IAA, L-tyrosine, hippuric acid, and ferulic acid sulphate) and semi-quantification of 201 nutrition biomarkers of fruits and vegetables (*N* = 105), meat (*N* = 8), fish (*N* = 3), seeds (*N* = 17), olive oil (*N* = 4), coffee (*N* = 10), curcuma (*N* = 2), garlic and onion (*N* = 1), grains (*N* = 9), soft drinks (*N* = 3), alcoholic beverages (*N* = 13), and other groups (i.e., potato, cocoa, mushrooms, legumes, and nuts; *N* = 26) (see Supplementary Table S1) was developed based on previous results [23]. A total of 110 µL of 1:20 (*v*/*v*) diluted urine samples in deionized water were added to a 96-well plate and mixed with 10 µL of IS (i.e., caffeine-D_9_, phenylalanine-D_5_, betaine-D_11_, and taxifolin at 15 µM each).

UHPLC-MS/MS analysis was conducted using a Sciex QTRAP 6500+ system (Sciex, Framingham, MA, USA) operating in positive and negative ionization modes (ESI+/−). Separations were performed using a Luna Omega Polar C18 column (100 mm × 2.1 mm, 1.6 µm) equipped with a fully porous polar C18 security guard cartridge (Phenomenex, Torrance, CA, USA). Conditions were as follows: column temperature, 40 °C; autosampler temperature, 10 °C; injection volume, 10 μL. Additionally, 0.1% formic acid and 10 mM ammonium formate in water and pure ACN were used as aqueous (A) and organic (B) mobile phases (MP), respectively. The gradient program was as follows: 0–8 min, 5–20% B; 8–10 min, 20–100% B; 10–12 min, 100% B; 12–12.1 min, 100–5% B; and 12.1–14 min, 5% B. MS detection was performed using the multiple reaction monitoring (MRM) mode (see Supplementary Table S2). The mass spectrometer operated using the following parameters: ion spray voltage, (±)4500 V; source temperature, 600 °C; curtain gas, 35 psi; ion source gases 1 and 2, 60 psi each; and entrance potential, (±)10 V. When available, the transitions were optimized by infusing 5 µM individual solutions of commercial standards dissolved in MP into the mass spectrometer. For the semi-quantification of nutrition biomarkers identified in urine samples for which authentic standards were not available, their corresponding transitions, as reported in the literature [23], were included (see Supplementary Table S1) and the semi-quantification was carried out using the relative peak areas. Quantification was carried out using the relative peak areas with an external calibration line using standard solutions obtained from serial dilutions of a working solution containing mixtures of pure analytical standards in ultrapure water. For monitoring the instrument’s performance, a quality control (QC) sample (5 µL of each sample pooled) was analyzed every 20 samples in the randomized analytical batch. The batch acceptance criterion was QC relative standard deviation (RSD) < 25%. In addition, calibration blanks (i.e., H_2_O) and a process blank (i.e., processing of H_2_O as described for urine samples) were injected at the beginning of the sequence for system suitability testing.

Urinary biomarker concentrations were normalized to creatinine concentrations quantified following the manufacturer’s instructions of the modified Jaffe’s method implemented in the DetectX^®^ urinary creatinine detection kit from Arbor Assays (Ann Arbor, MI, USA). Samples were diluted with deionized water prior to measurements employing a 1:20 (*v*/*v*) dilution.

### 2.5. Method Validation

Method validation was based on the US Food and Drug Administration (FDA) guidelines for bioanalytical method validation [38], including the following bioanalytical parameters: accuracy, precision, linearity range, carryover, selectivity, specificity, and stability.

Replicates (*N* = 3) of standards at three concentration levels and replicates (*N* = 3) of spiked samples at three concentration levels (low, medium, and high) on three validation days were analyzed to assess accuracy. The RSD of replicate standards/samples within one validation batch (intra-day) and between validation batches (inter-day) was used to estimate precision. The calibration curves included a zero calibrator (i.e., blank with IS) and, at least six standards covering the selected concentration ranges ensuring linearity. Carryover between samples was assessed by the analysis of a calibration blank after the injection of the standards. The analysis of calibration and process blank samples from multiple (*N* = 6) sources were used to demonstrate selectivity and specificity. Analytes’ freeze-thaw and long-term stabilities were tested by comparing concentrations observed in a freshly prepared sample to sample extracts after three freeze-thaw cycles and to a sample stored for six months (−80 °C).

### 2.6. Data Availability and Statistical Analysis

UHPLC-MS/MS data were acquired and processed using SCIEX OS Software (Sciex, Framingham, MA, USA). Data analysis was carried out in MATLAB R2019b (MathWorks, Natick, MA, USA) and using the PLS Toolbox 8.9 (Eigenvector Research Inc., Manson, WA, USA). Biomarker levels normalized to creatinine in urine samples determined in this work are available in Supplementary Table S3. Continuous variables were expressed as mean ± standard deviation or medians with interquartile range, depending on underlying data distribution. Student’s *t*-test (α = 0.05) or Wilcoxon rank-sum test (α = 0.05) were used for inter-group comparison. Pearson’s and Spearman’s correlation coefficients were used for assessing paired associations among metabolite concentrations, and among metabolite concentrations and R24h food groups, respectively. Hierarchical clustering analysis was carried out using autoscaled data.

Diversity of microbiota biomarkers within a sample was assessed with Simpson’s Index of Diversity (1−D), where *D* was defined as:

(1) $$D=\frac{\sum n(n-1)}{N(N-1)}$$

*n* being the concentration of a particular microbiota activity biomarker in the sample; and *N* the total concentration of microbiota activity biomarkers. Thus, the higher the value for this index (1−D), the higher the microbiota activity biomarker diversity.

## 3. Results and Discussion

### 3.1. UHPLC-MS/MS Method Validation

The recommended guidelines were used to perform the validation of the analytical method [38]. Linearity of response was assessed covering up to four orders of magnitude with limits of detection (LODs) and limits of quantification (LOQs) in the 0.03–8 μM range (see Supplementary Table S2) and carryover did not exceed 20% of LOQ. Regarding accuracy and precision, intra- and inter-day recoveries in standard solutions spiked at the different concentration levels were between 82 and 119%, with an average precision of 7% (min-max precision of 1–20%) (see Supplementary Table S4). Similarly, in spiked urine samples, intra-day and inter-day recoveries and precisions ranged between 80 and 120% and between 1 and 20% (average precision of 10%), respectively, with the exception of galactitol and genistein spiked at the low concentration level that presented an inter-day recovery of 123% and an inter-day accuracy %RSD of 21. This evidences an adequate method performance for all quantified metabolites. Additionally, all compounds were stable after freezing stock solutions for one year at −80 °C, and after three freeze-thaw cycles (−80 °C) (*t*-test, *p*-values > 0.05). Process blanks using sterile PP containers were analyzed and compared to calibration blanks to assess selectivity and specificity, as well as to test the compatibility of the sample collection procedure with the analysis of these metabolites, and no contaminations were detected.

### 3.2. R24h Results

**Figure 1:**
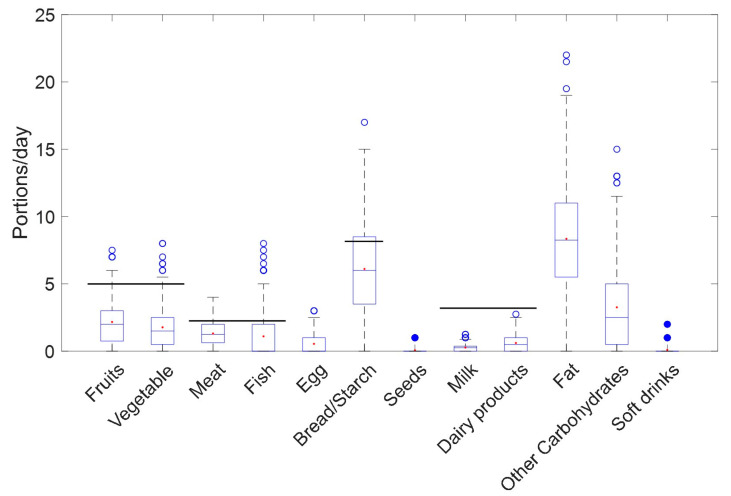
R24h food groups intake in lactating mothers. Note: Horizontal black line represents recommended intake according to the World Health Organization diet guidelines for lactating mothers. (Red dot = median; blue circle (open) = standard outlier (1.5 - 3.0 × IQR); blue circle (closed) = extreme outlier (≥ 3.0 × IQR)).

As shown in Figure 1, results from R24h evidence that mothers’ intake of fruits, vegetables, meat, milk, egg, fish, dairy, and other carbohydrates ranged between 1 and 5 portions/day, while bread/starch and fat were the food groups of higher intakes, with 5 to 10 portions/day. However, seeds, and soft drinks were rarely present in their diets. The majority of the mothers participating in the study reported lower intake of fruits and vegetables, meat and fish, bread/starch, and milk, and dairy products than the recommended intake according to the World Health Organization diet guidelines for lactating mothers (5, 2, 8.5, and 3 portions/day, respectively) [39]. Additionally, results from FFQ indicate that 51 of the 59 mothers (86%) included in the study presented adherence to the Mediterranean diet (i.e., MedDietScore between 25 and 35), while 8 women showed lower adherence (7 and 1 participants < 35 and <25, respectively).

### 3.3. Biomarker Profiles of Lactating Mothers

The concentrations of the nutrition and microbiota biomarkers determined in urine samples, after normalization to creatinine using the validated UHPLC-MS/MS method, are shown in Table 2.

Results show that, overall, median concentrations of microbiota biomarkers were relatively low, with the exception of hippuric acid and L-tyrosine which were higher than the rest. As observed, a high variability (two to four orders of magnitude) was encountered for these two microbiota biomarkers. It is noteworthy that 3-IPA, an end product of tryptophan metabolism, was only detected in 3% of the samples, whereas the other microbiota biomarkers were detected in over 62% of the samples. In respect of the targeted analysis of nutrition biomarkers, the highest concentrations were found for TMAO, proline betaine, taurine, and 1- and 3-methylhistidine, with phloretin, O-DMA, genistein, hesperetin, and glycitein being the least abundant BFIs. As with the microbiota biomarkers, broad concentration ranges for the more the prevalent metabolites were observed, while lower detection frequencies were found for certain less-abundant biomarkers (e.g., genistein, phloretin, and glycitein) were found.

**Figure 2:**
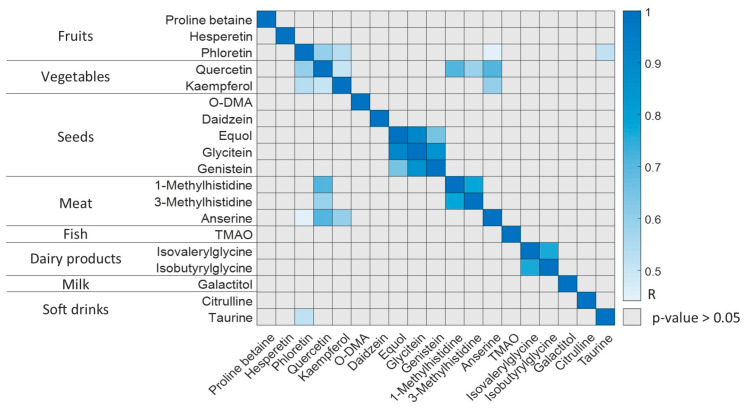
Significant paired correlations among BFIs (Pearson’s correlation, *p*-value < 0.05).

Figure 2 depicts the significant paired correlations among BFIs (*p*-value < 0.05). Some metabolites that are biomarkers of the same food group are significantly correlated among each other, such as 1- and 3-methylhistidine (meat); isovalerylglycine and isobutyrylglycine (dairy products); quercetin, phloretin and kaempferol (fruits and vegetables); and genistein, equol and glycitein (seeds). However, additional correlations between biomarkers from different food groups were also significant, such as meat BFI anserine with fruits and vegetables BFIs (i.e., quercetin, phloretin and kaempferol), soft drink BFI taurine with phloretin, and some meat BFIs (i.e., 1- and 3-methylhistidine) with quercetin. This could be due to the paired intake of both food groups or chance correlations.

**Figure 3:**
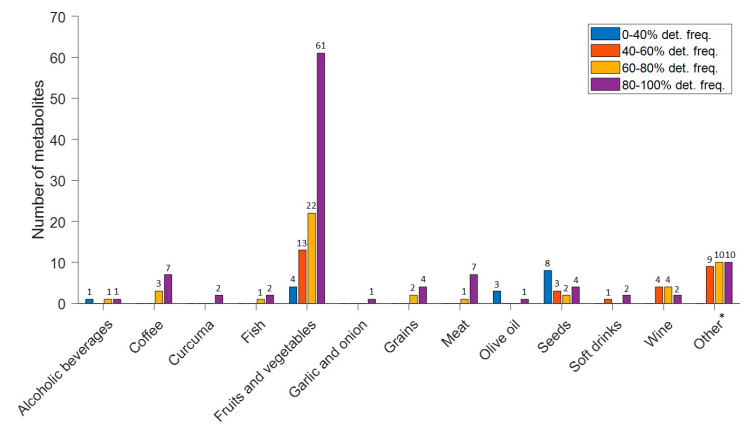
Number of metabolites and detection frequencies of the semi-quantitative analysis. Note: (*) Potato, cocoa, mushrooms, legumes and nuts.

Regarding the semi-quantitative analysis, a higher number of detected metabolites belonged to fruits and vegetables, followed by other groups (i.e., potato, cocoa, mushrooms, legumes, and nuts), coffee, meat, and seeds, as shown in Figure 3.

### 3.4. R24h and BFIs in Lactating Mothers

**Figure 4:**
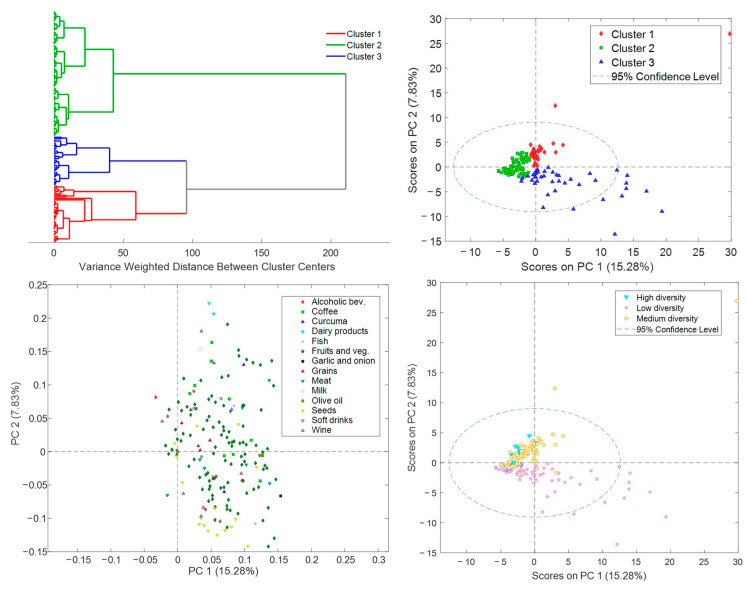
BFIs patterns in mothers’ urine samples. **Top**: Hierarchical clustering analysis revealing three sub-groups within the study samples (**left**) and PCA scores plot (**right**). **Bottom**: loadings plot (**left**) and PCA scores plot with Simpson’s Index of Diversity classes (**right**).

In Figure 4 (top, left), BFI patterns in mothers’ urine samples detected by hierarchical clustering analysis (HCA) are shown. Three clusters were identified as clusters 1, 2, and 3, and were depicted in the Principal Component Analysis (PCA) scores plot in Figure 4 (top, right). Samples from clusters 1 and 3 presented higher concentrations of all biomarkers than samples from cluster 2, while dairy product and milk biomarkers were more concentrated in cluster 1, and seeds and garlic and onion BFIs in cluster 3 as shown in the PCA loadings plot in Figure 4 (bottom, left).

**Figure 5:**
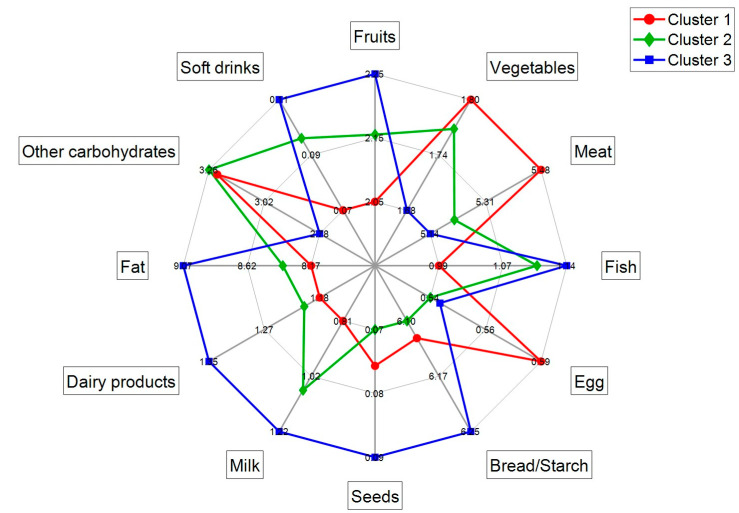
Mean R24h food group values for clusters 1 to 3.

Figure 5 depicts the mean R24h food group values for each of the three clusters detected by analysing BFI patterns in urine samples. It shows that mothers from cluster 1 had a higher intake of some food groups (i.e., vegetables, meat, egg, and other carbohydrates) than mothers from cluster 3, who presented a lower intake, evidencing that they followed a different dietary pattern. Conversely, mothers in cluster 3 had highest intakes of fruits, fish, bread/starch, seeds, milk, dairy products, fat, and soft drinks. Mothers from cluster 2 showed a mean intake in between mothers from clusters 1 and 3 for all food classes, except for other carbohydrates, egg, bread/starch, and seeds.

In concordance with the results from the HCA, clusters 1 and 3 present higher intakes of the majority of food groups and cluster 3 is characterized by a high intake of seeds. However, R24h results show that the class with lowest consumption of milk and dairy products is cluster 1, contradictory to the HCA results. These divergences could be due to the small absolute difference in the mean R24h food group values between clusters, as the majority of the mothers follow a similar Mediterranean diet (*p*-value > 0.05, Wilcoxon rank-sum test), and/or errors due to the subjective nature of the R24h recording. Additionally, we would like to highlight the complementary information provided by R24h food groups and BFIs, as the information provided by BFIs is more detailed and reports food groups which are not specified in R24h (e.g., garlic and onion, alcoholic beverages, coffee, curcuma, olive oil, wine). Furthermore, these differences hamper the comparison of the information accessible through both R24h and BFIs.

**Figure 6:**
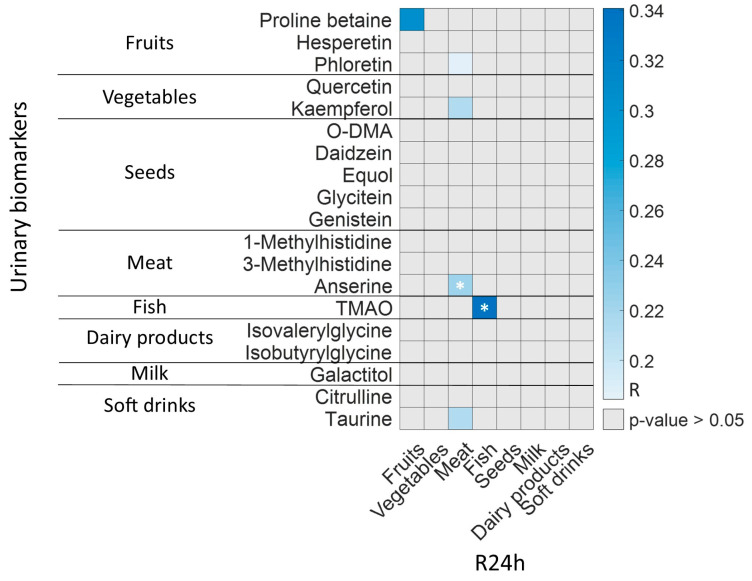
Significant correlations among BFIs and R24h food groups (Spearman’s correlation, *p*-value < 0.05). Note: (*) *p*-value < 0.001.

Correlations between urinary BFIs and the food groups obtained in the R24h are depicted in Figure 6, and show significant correlations between three biomarkers (i.e., proline betaine, anserine, and TMAO) and their corresponding food groups (i.e., fruits, meat, and fish) from the R24h, with a *p*-value < 0.01 for fruits, and *p*-values < 0.001 for meat and fish (*r* > 0.2, Spearman’s correlation). These results strengthen the findings of Lloyd et al., reporting urinary excretion of proline betaine as a potentially useful biomarker of habitual citrus consumption [40], and the conclusions reached by Cheung et al. in which anserine and TMAO are presented as potential biomarkers of chicken and fish intake, respectively [41]. Additionally, there are three other metabolites that are significantly correlated to meat (i.e., phloretin, kaempferol, and taurine), which are described as fruit, vegetable and soft drink BFIs [42,43]. This could be due to a paired intake of vegetables and meat (see Figure 5) and/or due to the correlation of taurine levels with meat intake reported in other studies, as ingestion of foods of animal origin can be another source of taurine [44,45,46]. It should be remarked that not all food groups recorded in the R24h have their corresponding urinary BFI (i.e., egg, bread/starch, fat and other carbohydrates), due to the difficulty in finding specific BFIs [47].

On the other hand, the specificity of most of the already proposed BFIs is limited, as many dietary compounds are present in different types of foods and similar compounds from different food sources can result in the same metabolite [47]. There are additional concerns, such as the lack of knowledge about the dose–response relationships, the limited quantitative data available, the interindividual variation that can lead to different BFIs levels with the same intake levels [47,48], and the fact that some metabolites are derived not only from diet, but also from other parallel endogenous pathways affected by intake [49]. However, self-reported surveys are known to be prone to misreporting issues and, therefore, can lead to uncertain research findings. Consequently, due to the high complexity of accurate dietary assessments, the implementation of a combined strategy bringing together complementary tools might allow enhanced performance to be achieved.

Despite the fact that the mothers included in the study are similar ages, the same diet could lead to different metabolite by-products due to differences in metabolism between individuals, as recent studies have found significant associations between food intake metabolites and body mass index (BMI) [50,51]. However, in this study we did not find any significant correlation between mothers’ BMI and their food intake (*p*-value > 0.05, Spearman’s correlation). In this study, repeated sampling from the same individual was performed, corresponding to the collection of urine samples and R24h at different study time points, but it has to be taken into consideration that lactating mothers are a particular population, as the body readjusts after giving birth, usually resulting in a weight reduction.

### 3.5. Microbiota Activity Biomarkers in Lactating Mothers

The diversity of microbiota activity biomarkers was estimated through the Simpson’s Index of Diversity (Equation (1)) and ranged between 0 and 0.6 with a median value of 0.08. Samples were classified as low (*N* = 111), medium (*N* = 60), and high (*N* = 37) diversity for Simpson’s Index of Diversity if they were under 0.05, between 0.05 and 0.25, and over 0.25, respectively. In Figure 4 (bottom, right), a PCA scores plot of the urinary BFIs concentrations with the microbiota activity biomarker diversity classes assigned is shown. Samples classified as high and medium diversity presented different BFI levels from the samples classified as low diversity, reflecting a nexus between dietary patterns and microbiota activity. This corroborates findings reported by Turnbaugh et al. indicating that diet has a significant impact on sculpting the microbial communities in the gut, and changes in dietary patterns can directly influence the composition and functionality of the gut microbiota through the availability of macro- and micronutrients in the gut [52]. More specifically, the high number of samples from dietary cluster 3 that show a low microbiota activity diversity (43%) is noticeable. However, BFIs and microbiome analysis should be assessed in more detail in further studies on the impact of diet on the gut microbiome.

## 4. Conclusions

A UHPLC-MS/MS method for the analysis of BFIs and microbiota activity biomarkers was developed and validated. Levels of microbiota activity biomarkers were reported and a panel of both quantitative and semi-quantitative BFIs was determined in an observational study involving lactating mothers. Correlations between some BFIs in the same food group were found (i.e., meat, dairy products, fruits and vegetables, and seeds). Additionally, the BFI profile allowed three clearly distinct dietary patterns present in the study population to be discerned, evidencing the capability of urinary food intake assessment as a complementary tool to traditionally employed questionnaires. The presence of some highly specific BFIs in urine samples (e.g., curcuma and garlic and onion) highlights its complementary nature. Correlations were found between fruit, meat, and fish biomarker concentrations and R24h results. This evidences that the use of some biomarker measurements for the assessment of some food group intakes is possible, even though more studies are needed in order to expand the number of food groups that can be assessed by BFIs. Furthermore, the diversity of microbiome activity biomarkers reflected dietary patterns detected in lactating mothers. In future studies, biomarkers and microbiome analysis will be integrated for a joint assessment of diet, microbiome, and, ultimately, health.

## Figures and Tables

**Table 1:** Demographic and clinical characteristics of participants at time point ‘Recovery of Birth Weight’ or ‘Complete Enteral Nutrition’ for mothers of term and preterm infants, respectively.

Parameters	Mothers (*N* = 59)
Age (years), mean (SD)	36 (5)
Weight (kg), mean (SD)	62 (14)
BMI (kg/m^2^), mean (SD)	24 (5)
C-Section delivery, N (%)	28 (48)
Antibiotic therapy, N (%)	3 (5)
Dietary supplements, N (%)	52 (88)
MedDietScore *, mean (SD)	30 (5)

Note: * derived from FFQ recorded at month one; SD = standard deviation; BMI = body mass index; C-Section = caesarean section; MedDietScore = adherence to the Mediterranean diet.

**Table 2:** Microbiota and nutrition biomarker concentrations in urine samples from lactating mothers.

Category	Metabolite	Range (µmol/g Creat)	Median (µmol/g Creat)	IQR (25–75)	Detection Frecuency (%)
Microbiota	Phenylpropionylglycine	0.007–58	0.03	0.4	72
	3-IPA	0.012–8	2	3	3
	L-Kynurenine	0.009–21	0.5	2	80
	3-IAA	0.012–176	1.3	7	99
	L-Tyrosine	0.014–2829	16	43	98
	Hippuric Acid	11–8496	255	598	100
	Gallic Acid	0.013–10	0.4	0.4	62
	Ferullic Acid Sulphate	0.02–126	0.4	3	74
Fruits	Proline betaine	1.4–2568	88	315	100
	Hesperetin	0.02–11	0.05	0.13	64
	Phloretin	0.004–2	0.02	0.10	29
Vegetables	Quercetin	0.03–76	0.4	0.2	48
	Kaempferol	0.02–2	0.2	0.6	93
Seeds	O-DMA	0.007–5	0.02	0.3	61
	Daidzein	0.005–3	0.4	0.9	96
	Equol	0.012–10	0.3	0.05	34
	Glycitein	0.006–1	0.05	0.2	25
	Genistein	0.4–2	0.03	0.02	2
Meat	1-Methylhistidine	4–1784	48	95	100
	3-Methylhistidine	0.2–4555	31	87	100
	Anserine	0.6–304	2	10	88
Fish	TMAO	3–3368	142	473	100
Dairy products	Isovalerylglycine	0.005–91	3	13	87
	Isobutyrylglycine	0.7–125	3	9	93
Milk	Galactitol	1.2–2424	22	67	92
Soft drinks	Citrulline	0.09–60	7	11	95
	Taurine	0.2–2341	55	207	100

Note: 3-IPA = 3-indolepropionic acid; 3-IAA = indole-3-acetic acid; O-DMA = O-desmethylangolensin; TMAO = trimethylamine N-oxide; IQR = interquartile range.

## Data Availability

The data presented in this study are available in supplementary material here.

## Supplementary Materials

The following supporting information can be downloaded at: https://www.mdpi.com/article/10.3390/nu15081894/s1, Table S1: Measurement parameters and main figures of merit of the semi-quantitative LC-MS method for BFI determination; Table S2: Measurement parameters and main figures of merit of the targeted LC-MS method for nutrition and microbiota biomarker determination; Table S3: Nutrition and microbiota biomarker levels in urine samples from lactating mothers; and Table S4: Calculated intra- and inter- day accuracy (i.e., recovery) and precision (i.e., RSD) of the LC-MS method in standard solutions and spiked urine samples.
